# Supplementary material for: KLK3/PSA and cathepsin D activate VEGF-C and VEGF-D
Source: eLife. 2019 May 17;8:e44478. doi: 10.7554/eLife.44478 (PMC6588350; doi:10.7554/eLife.44478)
Supplement: Supplementary file 1. — The data for the antibodies in this list were obtained from the product description provided by the supplier. Data missing from suppliers’ websites were obtained by direct request to customer support. The dilution refers to what was used for the Western blot analyses performed in this study and is identical to the supplier’s recommendation for commercially available antibodies. [file elife-44478-supp1.docx]

## Supplementary Table T1.

## List of anti-VEGF-C antibodies used in this study.

| **Antibody** | **Antigen** | **Purification** | **Supplier** | **Dilution** |
| --- | --- | --- | --- | --- |
| *Antisera (AS)* | | | | |
| AS no. 6 | histagged, human VEGF-C (aa 32-419) produced in HighFive cells | None | [(Baluk et al. 2005)](https://paperpile.com/c/mIUAVE/s0lG) | 1:2000 |
| AS 882 | peptide (aa 104-120): NH_2_-EETIKFAAAHYNTEILK = N-terminal sequence of minor, mature VEGF-C | None | [(Joukov et al. 1997)](https://paperpile.com/c/mIUAVE/g3IH) | 1:1000 |
| AS 905 | peptide (aa 33-54): NH_2_-ESGLDLSDAEPD AGEATAYASK = N-terminal sequence of the N-terminal propeptide | None | [(Joukov et al. 1997)](https://paperpile.com/c/mIUAVE/g3IH) | 1:500 |
| AS 885 | peptide (aa 120-136): NH_2_-KSIDNEWRKTQ CMPREV = N-terminal sequence of hypo- thetical shortest active form of mature VEGF-C | None | In-house | 1:250 |
| AS 890 | peptide (aa 372-394): NH_2_-KGKKFHHQTCS CYRRPCTNRQKA = internal peptide close to the C-terminus of the silk homology domain | None | In-house | 1:250 |
| AS no. 3/4 | histagged, human VEGF-C (aa 103-215) produced in HighFive cells | None | In-house | 1:1000 |
| *Polyclonal antibodies* | | | | |
| [ab9546](https://www.abcam.com/vegfc-antibody-ab9546.html) | rat VEGF-C protein (aa 101-221) produced in insect cells | protein A-purified | Abcam | 1:1000 |
| [ab135506](https://www.abcam.com/vegfc-antibody-ab135506.html) | recombinant human VEGF-C protein (aa 1-290) | protein A and peptide affinity-purified | Abcam | 1:1000 |
| [NB110-61022](https://www.novusbio.com/products/vegf-c-antibody_nb110-61022) | purified, recombinant rat VEGF-C protein (aa 101-221) | protein G purified | Novus (Biotechne) | 1:1000 |
| [#2445](https://www.cellsignal.com/products/primary-antibodies/vegf-c-antibody/2445) | peptide (residues surrounding Thr189 of human VEGF-C) | protein A and peptide affinity-purified | Cell Signaling Technology | 1:1000 |
| [PA5-29772](https://www.thermofisher.com/antibody/product/VEGFC-Antibody-Polyclonal/PA5-29772) | human VEGF-C protein (aa 180-414) | antigen affinity purified | Invitrogen/ ThermoFisher | 1:500 |
| [SAB1303101](https://www.sigmaaldrich.com/catalog/product/sigma/sab1303101) | recombinant human VEGF-C protein (aa 1-290) | affinity-purified | Sigma-Aldrich/ Merck | 1:500 |
| [SAB1303607](https://www.sigmaaldrich.com/catalog/product/sigma/sab1303607) | a synthetic peptide located between aa 248-277 | affinity-purified | Sigma-Aldrich/ Merck | 1:500 |
| [AF752](https://www.rndsystems.com/products/human-vegf-c-antibody_af752) | recombinant human VEGF-C produced in *E. coli* (aa 104-330) | antigen affinity-purified | R&D Systems (Biotechne) | 1:1000 |
| *Monoclonal antibodies* | | | | |
| [sc-374628](https://www.scbt.com/scbt/product/vegf-c-antibody-e-6) | peptide (aa 103-137): NH_2_-TEETIKFAAAHY NTEILKSIDNEWRKTQCMPREVC =  N-terminus of minor, mature VEGF-C | protein A-purified | Santa Cruz Biotechnology | 1:500 |
| [sc-101583](https://www.scbt.com/scbt/product/vegf-c-antibody-mm0006-2e65) | raised against recombinant human VEGF-C protein (aa ???-???) | protein A-purified | Santa Cruz Biotechnology | 1:500 |
| [MAB752](https://www.rndsystems.com/products/human-vegf-c-antibody-193208_mab752) | recombinant human VEGF-C protein (C156S mutant, aa 103-227) produced in the mouse myeloma cell line NS0 | protein A/G purified from hybridoma sup | R&D Systems (Biotechne) | 1:500 |
| [MA5-26494](https://www.thermofisher.com/antibody/product/VEGFC-Antibody-clone-OTI4A1-Monoclonal/MA5-26494) | recombinant major, mature form of human VEGF-C (aa 112-227) produced in *E. coli* | affinity purified | Invitrogen/ ThermoFisher | 1:200 |
| [SAB1306762](https://www.sigmaaldrich.com/catalog/product/sigma/sab1306762) | recombinant human VEGF-C protein (aa 1-290) | purified from hybridoma | Sigma-Aldrich/ Merck | 1:100 |
